# The mediating and moderating role of psychological resilience between occupational stress and mental health of psychiatric nurses: a multicenter cross-sectional study

**DOI:** 10.1186/s12888-022-04485-y

**Published:** 2022-12-23

**Authors:** Shu-Yan Chen, Shi-Rui Yan, Wei-Wei Zhao, Ying Gao, Wei Zong, Cheng Bian, Yin Cheng, Yan-Hong Zhang

**Affiliations:** 1 School of Nursing, Nanjing Medical University, Nanjing, China; 2 The Affiliated Brain Hospital of Nanjing Medical University, Nanjing, China

**Keywords:** Psychiatric nurses, Mental health, Occupational stress, Psychological resilience, Dual-factor model of mental health

## Abstract

**Background:**

The particular occupational stress of psychiatric nurses has a negative impact on their mental health. There is evidence that psychological resilience can promote mental health; however, the relationship between resilience in occupational stress and mental health of psychiatric nurses is unclear, and mental health was assessed from a psychopathological perspective, neglecting the role of positive psychology. Therefore, this study was based on a dual-factor model of mental health, describing mental health in terms of both positive well-being and psychiatric symptoms. We aimed to investigate the level of psychiatric nurses' mental health and whether resilience plays a mediating or moderating role between occupational stress and mental health in psychiatry nurses.

**Methods:**

A cross sectional survey of 450 psychiatric nurses in five hospitals in Jiangsu Province was conducted using a convenience sampling method, of which 413 were valid questionnaires with an effective rate of 91.8%. The evaluation included the Chinese Nurses’ Stress Scale, the Connor-Davidson Resilience Scale, the Warwick-Edinburgh Mental Well-being Scale, and the General Health Questionnaire. Descriptive and Spearman correlation analyses were performed using SPSS25.0 while mediating and moderating effects were performed using SmartPLS3.0.

**Results:**

Based on a dual-factor model of mental health, this study found that psychiatric nurses had a low general state of mental health, with 54.5% positive mental health, 7% vulnerable, 21.8% symptomatic but content, and 16.7% completely troubled. In addition, we found that resilience plays a mediating role in stress and mental health [β = -0.230, 95% *CI* of (-0.310, -0.150)] and does not play a moderating role [β = -0.018, 95%*CI* (-0.091, 0.055)].

**Conclusions:**

Psychiatric nurses are in a poor state of mental health, and psychological resilience partly mediates occupational stress and mental health. This study suggests that attention should be paid to both positive and negative aspects of psychiatric nurses' mental health, and strategies should be developed to reduce occupational stress and develop psychological resilience.

## Introduction

Nurses are recognized as having high-stress and high-risk careers [1], and occupational stress affects nurses' mental health, job satisfaction, and quality of life [2, 3]. Nurses, as important components of the healthcare system, spend most of their time providing direct care to patients. With multiple stressors [4–6] such as high clinical workload, inadequate respect and recognition, strained nurse-patient relationships, and discordant staff relationships, nurses are often overworked and overstressed, facing higher risks of suffering from anxiety and depression. In addition, for psychiatric nurses, the working situation is more severe due to patients with mental disorders, the closed working environment and the frequent occurrence of violence in the workplace [7–10]. Psychiatric nurses tend to have more prominent mental problems when they are in this stressful working environment for a long time [11]. It is noted that the prevalence of depression among psychiatric nurses in China and Australia is 36.6% [12] and 52.7% [6], respectively, indicating a poor level of mental health among psychiatric nurses. Therefore, the attention to the mental health of psychiatric nurses is a crucial issue to improve the current healthcare environment.

Psychological resilience describes the ability of an individual to adapt when faced with stress or difficulties, focusing on positive attitudes and strengths in the face of difficulties [13]. Psychological resilience is a protective factor that helps nurses cope with occupational stress [14]. When faced with the same pressures and challenges, nurses with high psychological resilience are better able to cope and achieve their growth and professional development [15]. As a result, psychological resilience is important in promoting mental health and improving the safety and quality of care.

Carson and Kuipers [16] have proposed three levels of the stress process, including stressors, moderators, and stress outcomes. The model suggests that stressful life events as stressors can influence mental health through seven factors such as resilience, social support, and self-esteem, and has been studied to confirm that psychological resilience plays a mediating role in stress and mental health [17]. The protective factor model of resilience theory [18] has stated that protective factors can decrease the cumulative effect of risk factors on negative outcomes and act as a buffer. Research also confirmed the moderating role of psychological resilience between stress and mental health [19]. Furthermore, there is research [20] that finds that psychological resilience plays a mediating and moderating effect between stress and mental health. In addition, although there has been an increase in research on psychological resilience in occupational stress and mental health in nursing, previous studies have mostly been conducted on nurses in general hospitals, and relatively few on psychiatric nurses, so it is unclear whether psychological resilience plays a mediating role, a moderating role, or both a mediating and moderating role in occupational stress and mental health. Therefore, it is necessary to explore the mechanisms of psychological resilience in occupational stress and the mental health of psychiatric nurses.

Mental health has often been evaluated from a psychopathological perspective, ignoring the potential and strengths of the individual. The dual-factor model of mental health emphasizes that mental health should be assessed from both positive and negative indicators to have a more comprehensive understanding of mental health status [21]. Therefore, based on the above theories, this study used occupational stress as an independent variable, psychological resilience as an intermediate variable, mental health described in both positive and negative dimensions as a dependent variable, and proposed two hypotheses, first, that psychological resilience plays a mediating role between occupational stress and mental health of psychiatric nurses, second, that psychological resilience plays a moderating role in occupational stress and mental health.

## Methods

### Study design and ethical considerations

A cross-sectional survey was conducted in August 2020 using a convenience sampling method to select psychiatric nurses from five hospitals in Jiangsu province. This study was reviewed and approved by the ethics committee (2021-KY112-01), and all data collected were confidential.

### Participants

Based on the minimum sample size recommended by Kline [22] which should be 10–20 times the number of each estimated parameter, the final sample size for this study was 144–325, taking into account the 10%-15% invalid questionnaires. A total of 450 questionnaires were issued and collected. After eliminating 37 invalid questionnaires, 413 valid questionnaires were received, with a valid recovery rate of 91.8%.

Inclusion criteria: acquired nursing license and within the valid registration period; with ≥ 6 months of psychiatric work experience; informed consent and voluntary participation. Exclusion criteria: not working in the hospital during the survey period, such as long-term sick leave or maternity leave; with major illnesses.

### Measures

#### General demographic information

General demographic information included gender, age, hospital grade, employment form, years as a nurse, educational experience, professional title, marital status, and the number of night shifts per week.

#### Independent variable: occupational stress

The Chinese Nurses’ Stress Scale(CNSS) [23] was used to measure occupational stress of psychiatric nurses. This scale consists of 5 dimensions and 35 items: 7 items for nursing profession and work, 5 items for workload and time allocation, 3 items for resource and environment problems, 11 items for patient care, and 9 items for management and interpersonal relationships. This scale is based on a 4-point Likert scale, with higher individual scores indicating higher levels of stress. The Cronbach's alpha coefficient of this scale in this study was 0.966, and the Cronbach's alpha coefficients of the subscales ranged from 0.854 to 0.936.

#### Intermediate variable: psychological resilience

The Chinese version of the Connor-Davidson Resilience Scale (CD-RISC) [24] was used to measure psychological resilience. The scale includes 3 dimensions of strength (8 items), optimism (4 items), and resilience (13 items), with a total of 25 items. The scale uses a Likert 5-point scale, with each entry ranging from 1 (never) to 5 (always). Scores range from 25 to 125, with higher scores indicating better psychological resilience. The Cronbach's alpha coefficient for this scale in this study was 0.957, with Cronbach's alpha coefficients ranging from 0.684 to 0.936 for each subscale.

#### Dependent variable: mental health

Positive well-being was measured using the Chinese version of the Warwick-Edinburgh Mental Well-being Scale (WEMWBS) [25]. There are 14 items, each scored on a 5-point Likert scale from 1 (never) to 5 (always), with a total score range of 14 to 70. Higher scores indicate higher levels of overall well-being. A score of 40 was used as a cutoff value to classify the level of positive well-being, with scores ≤ 40 indicating lower levels of well-being and others as high well-being [26]. The Cronbach's alpha coefficient for this scale in the study was 0.947.

Psychopathological symptoms were assessed using the Chinese version of the 12-item General Health Questionnaire(GHQ-12) [27]. This scale is one of the most commonly used instruments to measure mental health problems and can be used as a typical representative of negative indicators of mental health [28]. There are 12 items, including three dimensions, namely somatic symptoms (4 items), anxiety and worry (4 items), and depression (4 items). The scale is scored on a scale of 0–0-1–1 with a total score of 0–12, and a total score ≥ 3 is considered to have some degree of psychological problems [29]. In the present study, Cronbach's alpha coefficient for this scale was 0.890.

### Data collection

In this study, online data will be collected from five hospitals, including one primary psychiatric hospital (140 beds and 30 psychiatric nurses), three secondary psychiatric hospitals (three hospitals with a total of 1500 beds and 420 psychiatric nurses), and one tertiary psychiatric hospital (530 beds and 220 psychiatric nurses). The contact information of psychiatric nurses was obtained through the department of nursing in the hospital, and the QR code of the electronic questionnaire was sent after informing the purpose, significance, the principle of anonymity, and the inclusion and exclusion criteria of the study. Anonymity, voluntary principle, and inclusion and exclusion criteria were again indicated on the first page of the electronic questionnaire. If participants clicked on the online link and filled out the survey, consent was regarded to have been gained.

### Data analysis

This study used SPSS 25.0 and SmartPLS3.0 [30] for statistical analysis of the data. Descriptive analysis was used to describe the demographic characteristics and main variables of psychiatric nurses. The skewness and kurtosis are used to determine whether the main variables adhere to a normal distribution. If the variables have a normal distribution, the mean and standard deviation are used; otherwise, the median is used. Additionally, we performed a Spearman correlation analysis to explore the association of the variables with statistical significance at *p* < 0.05. Since mental health was measured in this study using WEMWBS and GHQ-12, which are formative measures, we used the partial least square structural equation model (PLS-SEM) to establish and test structural equation models to analyze the mediating and moderating effects of psychological resilience in the relationship between occupational stress and mental health. PLS-SEM analysis strictly follows a two-step approach, that is, checking the measurement and the structural models. The fit indices of the measurement model include Cronbach's alpha, composite reliability (CR), average variance extracted (AVE), loadings, variance inflation factors (VIF), indicator weights, and t-values. The fit indices of structural models involve R^2^, Q^2^ and standardized root mean residual (SRMR).

## Results

### Demographic information of psychiatric nurses

As detailed in Table 1, a total of 413 psychiatric nurses were included in this study with a mean age of 32.52 years and a mean year of experience of 11.14 years. Most of the nurses were female (90.1%) and married (72.9%). In China, hospitals are graded into three levels: tertiary, secondary and primary, with tertiary hospitals being the best, followed by secondary hospitals and finally primary hospitals. The majority of nurses were from secondary and tertiary hospitals, and only 17 were from primary hospitals. Only four nurses were employed in the form of personnel agency, the rest were in the authorized strength and contract system.

**Table 1:** Sociodemographic characteristics of psychiatric nurses (*N* = 413)

Characteristics	n	%	Mean ± SD	Range
Gender				
male	40	9.69%		
female	373	90.31%		
Age(years)			32.52 ± 7.264	21–61
≤ 30	192	46.49%		
31–40	177	42.86%		
41–50	26	6.29%		
> 50	18	4.36%		
Hospital-grade				
primary hospitals	17	4.12%		
secondary hospitals	260	62.95%		
tertiary hospitals	136	32.93%		
Employment form				
authorized strength	196	47.46%		
contract system	213	51.57%		
personnel agency	4	0.97%		
Years as a nurse			11.14 ± 7.87	0–36
≤ 3	51	12.35%		
4–10	193	46.73%		
≥ 11	169	40.92%		
Educational experience				
college degree and below	79	19.13%		
undergraduate degree and above	334	80.87%		
Professional title				
nurse	73	17.67%		
senior nurse	202	48.91%		
supervisor nurse	120	29.06%		
co-chief nurse and above	18	4.36%		
Marital status				
unmarried	107	25.91%		
married	302	73.12%		
divorced	4	0.97%		
Number of night shifts per week			2.53 ± 1.12	
0 per week	94	22.76%		
1–2 times per week	272	65.86%		
3 times and more per week	47	11.38%		

### Scores of CNSS, CD-RISC, WEMWBS and GHQ-12

Our results showed that psychiatric nurses had a total occupational stress score of (103.29 ± 26.31), a total psychological resilience score of (79.35 ± 15.68), and a total positive well-being score of (46.31 ± 9.18). Scores of the GHQ-12, which measure negative psychiatric symptoms, do not follow a normal distribution and are therefore described using a percentile, with a median of 2. The detailed information is shown in Table 2.

**Table 2:** Mean and standard deviation or percentiles of each variable (*N* = 413)

Number items	Min	Max	Mean	Standard deviation	Skewness	Standard deviation of Skewness	Kurtosis	Standard deviation of Kurtosis
**CNSS**	35	175	103.29	26.31	-0.076	0.120	0.021	0.240
professional & career issues	7	35	22.02	5.92	0.003	0.120	-0.116	0.240
workload & time pressure	5	25	17.04	4.61	-0.300	0.120	-0.117	0.240
resource & environment	3	15	9.32	3.18	-0.146	0.120	-0.434	0.240
patient care & interaction	11	55	32.26	8.52	0.022	0.120	0.349	0.240
interpersonal relationships & management	9	45	22.65	8.14	0.238	0.120	-0.293	0.240
**CD-RISC**	35	125	79.35	15.68	0.302	0.120	0.066	0.240
tenacity	14	65	40.01	8.61	0.363	0.120	0.205	0.240
strength	10	40	26.71	5.38	0.148	0.120	-0.116	0.240
optimism	4	20	12.63	2.72	0.205	0.120	0.130	0.240
**WEMWBS**	17	70	46.31	9.18	0.102	0.120	0.164	0.240
**Number items**	**Min**	**Max**	**Median**	**Percentile 25, Percentile 25**	**Skewness**	**Standard deviation of Skewness**	**Kurtosis**	**Standard deviation of Kurtosis**
**GHQ-12**	0	12	2	0, 4	1.264	0.120	0.620	0.240

*CNSS* Chinese Nurses’ Stress Scale, *CD-RISC* Chinese version of the Connor-Davidson Resilience Scale, *WEMWBS* Chinese version of the Warwick-Edinburgh Mental Well

According to the dual-factor model of mental health, the state of mental health of psychiatric nurses was classified into four groups based on high or low levels of positive well-being and the presence or absence of psychological symptoms, which were a. “positive mental health”, characterized by high well-being and low psychological symptoms; b. “vulnerable”, characterized by low well-being and low psychological symptoms; c. “symptomatic but content”, characterized by high well-being and high psychological symptoms; and d. “troubled”, characterized by low well-being and high psychological symptoms. As shown in Table 3, of the 414 psychiatric nurses, those with positive mental health accounted for 54.5%, those with low well-being and low psychological symptoms made up 7%, those with symptomatic but content 21.8%, and those with troubled 16.7%.

**Table 3:** Mental health status groups of psychiatric nurses according to the dual-factor model of mental health (*N* = 413)

Psychopathology	Positive wellbeing		Total
	**High**	**Low**	
**Low**	Positive mental health 225(54.5%)	Vulnerable 29(7%)	254(61.5%)
**High**	Symptomatic but content 90(21.8%)	Troubled 69(16.7%)	159(38.5%)
**Total**	315((76.3%)	98(23.7%)	

### Analysis of the correlation between CNSS, CD-RISC, WEMWBS, and GHQ-12

The results of Spearman's correlation analysis showed that occupational stress was negatively correlated with psychological resilience and positive well-being (*r* = -0.331, -0.444, both *P* < 0.001) and positively correlated with psychopathological symptom (*r* = 0.274, *P* < 0.001); psychological resilience was positively correlated with positive wellbeing (*r* = 0.786, *P* < 0.001) and negatively correlated with psychopathological symptom (*r* = -0.448, *P* < 0.001), see Table 4.

**Table 4:** The correlation of variables in the study (*N* = 413)

**Variable**	**CNSS**	P&CI	W&TP	R&E	PC&I	IR&M	**CD-RISC**	tenacity	strength	optimism	**WEMWBS**	**GHQ-12**
**CNSS**	1.000											
NP&W	0.804^**^	1.000										
W&TA	0.820^**^	0.691^**^	1.000									
R&E	0.795^**^	0.596^**^	0.682^**^	1.000								
PC	0.916^**^	0.647^**^	0.704^**^	0.699^**^	1.000							
M&IR	0.848^**^	0.544^**^	0.553^**^	0.609^**^	0.759^**^	1.000						
**CD-RISC**	-0.331^**^	-0.262^**^	-0.239^**^	-0.239^**^	-0.320^**^	-0.315^**^	1.000					
tenacity	-0.280^**^	-0.245^**^	-0.208^**^	-0.203^**^	-0.266^**^	-0.250^**^	0.945^**^	1.000				
strength	-0.355^**^	-0.258^**^	-0.241^**^	-0.245^**^	-0.352^**^	-0.355^**^	0.930^**^	0.803^**^	1.000			
optimism	-0.258^**^	-0.210^**^	-0.202^**^	-0.193^**^	-0.242^**^	-0.249^**^	0.845^**^	0.710^**^	0.784^**^	1.000		
**WEMWBS**	-0.444^**^	-0.347^**^	-0.344^**^	-0.294^**^	-0.412^**^	-0.417^**^	0.786^**^	0.722^**^	0.766^**^	0.669^**^	1.000	
**GHQ-12**	0.274^**^	0.262^**^	0.224^**^	0.166^**^	0.263^**^	0.230^**^	-0.448^**^	-0.401^**^	-0.429^**^	-0.416^**^	-0.470^**^	1.000

*CNSS* Chinese Nurses’ Stress Scale, *NP&W* Nursing profession and work, *W&TA* workload and time allocation, *R&E* Resource and environment problems, *PC* Patient care, *M&IR* Management and interpersonal relationships, *CD-RISC* Chinese version of the Connor-Davidson Resilience Scale, *WEMWBS* Chinese version of the Warwick-Edinburgh Mental Well-being Scale, *GHQ-12* 12-item Chinese version of the General Health Questionnaire

^**^ At the .001 level (double tail), the correlation is significant

### Evaluation of the measurement model

Evaluation of the measurement model is required before validating the structural model. Reliability and validity analyses were performed using the PLS algorithm. Since this study contains both reflective and formative constructs, we first examined the reflective construct, which involves Cronbach's alpha, CR, AVE and loadings. Cronbach's α and CR of all constructs in this study were > 0.9, suggesting a reliable internal consistency. The loading and AVE reflect the convergence validity of the model, and the values of loading and AVE in this study are greater than 0.7 and 0.5, respectively, indicating a good convergence of the constructs [31]. For formative indicators, CR, AVE, and other indicators are not applicable but are evaluated based on VIF, indicator weights, and their respective t-values. In this study VIF < 5, indicating that there is no significant covariance [31]. The indicator weights and the respective t values of the formative structure were significant for all indicators (t > 1.96, p < 0.05), as shown in Table 5. Both reliability and convergent validity indicate that the result of our measurement model is good and has a certain degree of reliability.

**Table 5:** Internal consistency reliability and convergent validity

Latent variables	Scale type	Indicator	Loadings	CR	Cronbach´s alpha	AVE	t-value	weight	VIF
Occupational Stress	Reflective	nursing profession and work	0.833	0.934	0.911	0.738	NA	NA	NA
		workload and time allocation	0.867				NA	NA	NA
		resource and environment problem	0.837				NA	NA	NA
		patient care	0.913				NA	NA	NA
		Management and interpersonal relationships	0.843				NA	NA	NA
Psychological resilience	Reflective	tenacity	0.928	0.949	0.919	0.861	NA	NA	NA
		strength	0.952				NA	NA	NA
		optimism	0.903				NA	NA	NA
Mental health	Formative	WEMWBS	NA	NA	NA	NA	48.028	0.934	1.250
		GHQ-12	NA	NA	NA	NA	3.768	-0.132	1.250

*CR* Composite reliability, *AVE* Average Variance Extracted (convergent validity), *NA* Not applicable, *VIF*: Variance Inflation Factor, t -values are 2.57, 1.96 and 1.65 for significance levels of 1%, 5% and 10%, respectively (two tailed tests)

### Evaluation of the structural model

After completing the evaluation of the measurement model, we further assess the structural model. R^2^ indicates the strength of each structural path and its value should be greater than or equal to 0.1 [32]. In our study, the results in Table 6 indicated that all R^2^ values were greater than 0.1. In addition, the Q^2^ for psychological resilience and mental health in this study were 0.085 and 0.428, respectively, which exceeded the recommended [33]. The predictive relevance is small when Q^2^ is 0.02, moderate when Q^2^ is 0.15, and large when Q^2^ is 0.35. Therefore, the predictive relevance of psychological resilience is small, while the predictive relevance of mental health is large. Moreover, the SRMR ≤ 0.1 in this study suggests that the model fit is acceptable [34].

**Table 6:** The Results of hypotheses

1. Mediation of psychological resilience between occupational stress and mental health(H1)				
**Relationship**	**Path coefficient(β)**	**t-Statistics**	***P-value***	**95%CI**
OS—> PR	-0.318	5.983	0.001	-0.422, -0.212
PR—> MH	0.725	23.670	0.001	0.660, 0.782
OS—> PR—> MH (indirect effect)	-0.230	5.673	0.001	-0.310, -0.150
OS—> MH (direct effect)	-0.219	5.910	0.001	-0.295, -0.149
	**R**^**2**^	**Q**^**2**^	**SRMR = 0.046**	
PR	0.101	0.085		
MH	0.675	0.428		
**2. Moderation of psychological resilience between occupational stress and mental health(H2)**				
**Relationship**	**Path coefficient(β)**	**t-Statistics**	**P-value**	**95%CI**
OS—> MH	-0.216	6.193	0.001	-0.287, -0.150
PR—> MH	0.723	22.580	0.001	0.658, 0.783
OS*PR- > MH	-0.018	0.492	0.622	-0.091, 0.055
	**R**^**2**^	**Q**^**2**^	**SRMR = 0.046**	
MH	0.675	0.424		

*OS* Occupational stress, *PR* Psychological resilience, *MH* Mental health, *R*^*2*^ R square, *Q*^*2*^ construct cross validated redundancy, *CI* Confidence interval

### The mediating role of psychological resilience

**Fig. 1:**
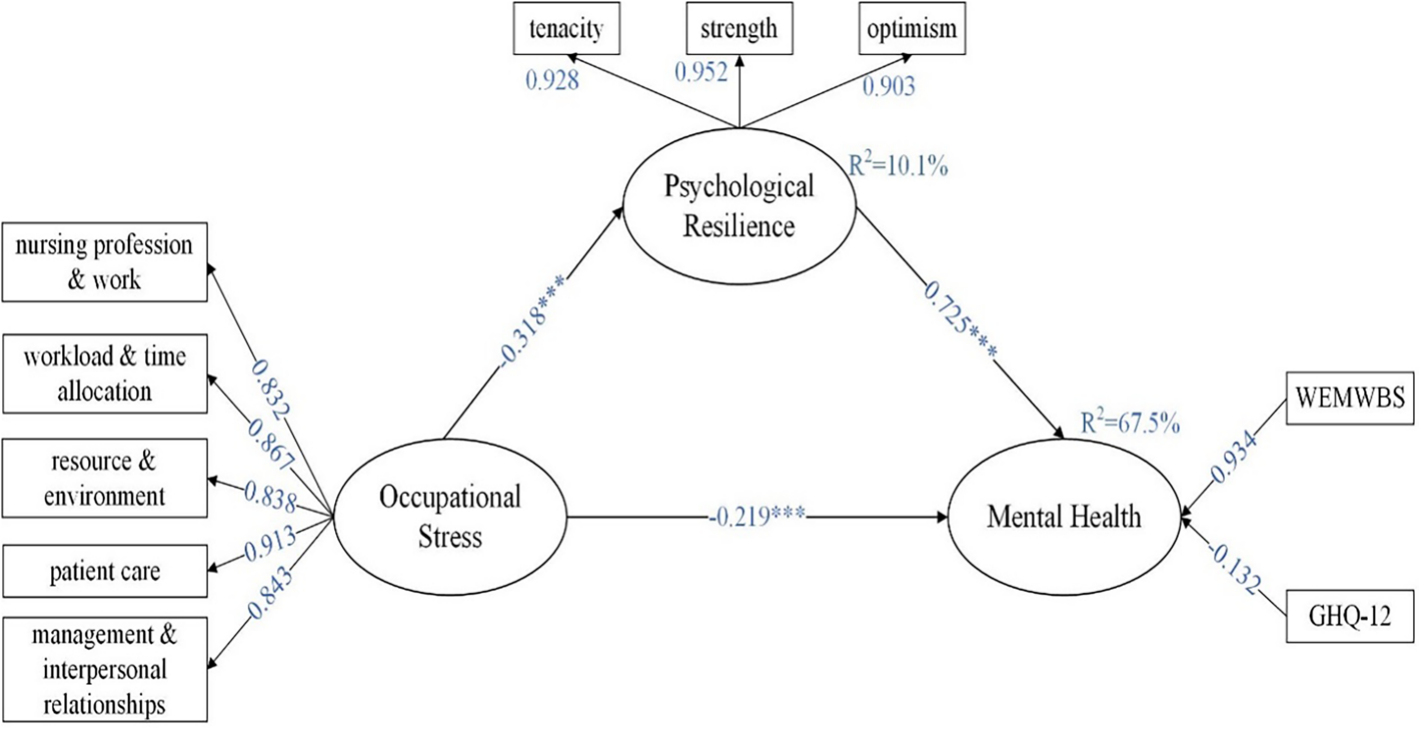
Mediation of psychological resilience

Our study put psychological resilience as a mediating variable in the model, thus exploring the mediating role of psychological resilience between occupational stress and mental health. The bootstrapping repeated sampling method was used to sample 5000 times to obtain the path coefficients and significance t-values between each variable. T-values greater than 1.96 indicate significant differences in the results. As shown in Table 6 and Fig. 1 titled “Mediation of psychological resilience”, the results revealed that the path coefficient of the mediating effect of occupational stress on mental health through psychological resilience β = -0.230 with a 95% *CI* of (-0.310, -0.150) excluding 0, while the path coefficient of the direct effect of occupational stress on mental health β = -0.219, p < 0.01. This indicates that psychological resilience plays a partially mediating role, supporting our research hypothesis 1. In addition, the R^2^ for psychological resilience and mental health were 0.101 and 0.675, respectively, indicating that the model explained 10.1% of the variance in psychological resilience and 67.5% of the variance in mental health.

### The moderating role of psychological resilience

**Fig. 2:**
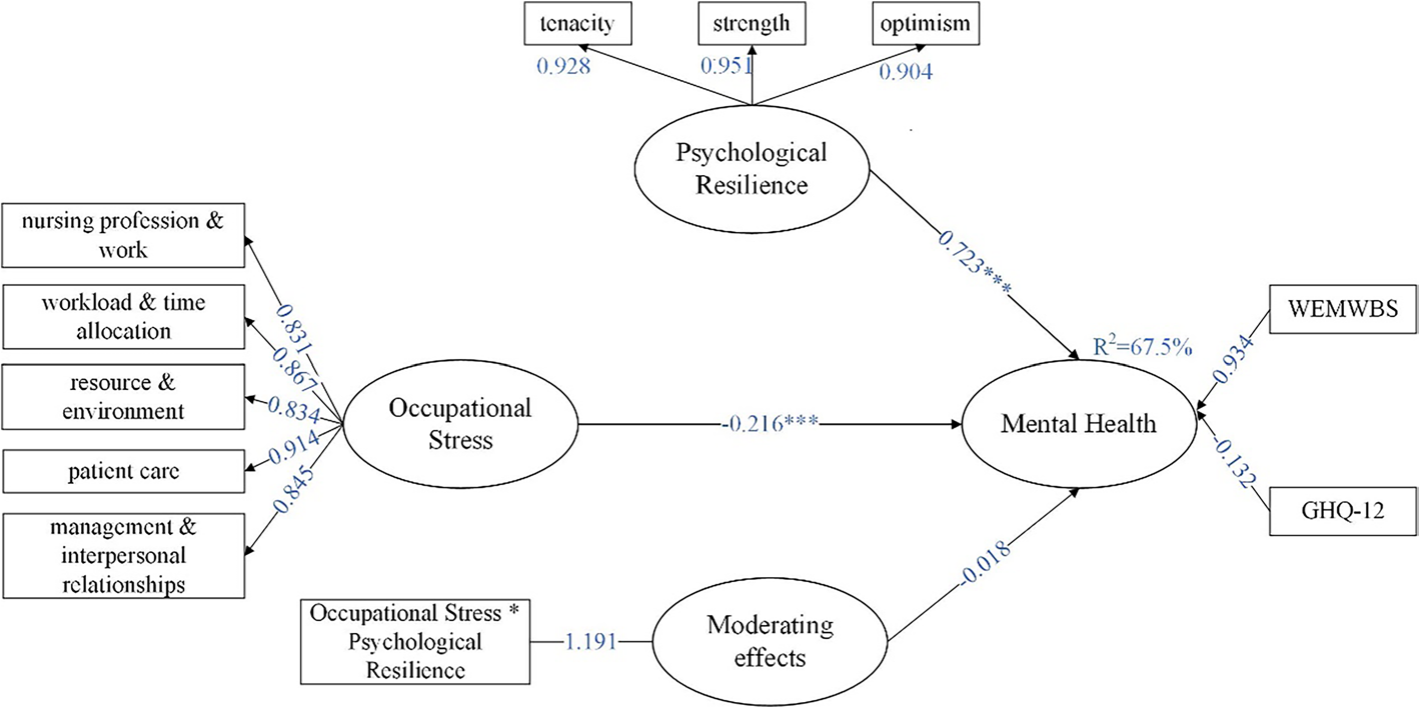
Moderation of psychological resilience

Psychological resilience was used as a moderating variable to determine whether psychological resilience plays a moderating role between occupational stress and mental health by interacting psychological resilience with latent variables in the model and exploring the interactive effect on mental health. The results indicated that the interaction effect was not significantly associated with mental health [β = -0.018, 95%*CI* (-0.091, 0.055)], indicating that psychological resilience did not play a moderating role, as shown in Table 6 and Fig. 2 titled “Moderation of psychological resilience”.

## Discussion

Our results showed that the occupational stress score of psychiatric nurses was (103.29 ± 26.31), which was similar to CNSS score of (99.29 ± 9.96) for psychiatric nurses in another study in China [35], but significantly higher than the CNSS score of (88.6 ± 21.0) with Chinese community nurses [36]. This suggests that the occupational stress of psychiatric nurses is relatively high. High levels of occupational stress may be related to the patients they care for and the environment in which psychiatric nurses work. Since psychiatric nurses provide care for patients with mental disorders, caring for such patients is inherently difficult and challenging, and this complexity can lead to greater stress for nurses [37]. The non-enclosed work environment is one of the reasons for the increased occupational stress of nurses. In addition, the anxiety, panic and fear of being infected during the COVID-19 pandemic put nurses under higher pressure to provide nursing care than in previous years. In addition, workplace violence is more likely to occur during the COVID-19 pandemic, which can also have an impact on nurses' occupational stress.

Mental health is a state-related to mental health and psychological well-being, and the positive dimension of mental health cannot simply be considered as the absence of negative symptoms. It is more comprehensive to measure mental health in both positive and negative dimensions [38]. In this study, positive well-being of psychiatric nurses as measured using the WEMWBS and the total WEMWBS score was (46.31 ± 9.18), which was significantly lower than the WEMWBS score of Chinese medical staff (56.05 ± 9.56) [39]. In addition, the WEMWBS score in this study is similar to the UK Mental Health Nurses score (47.57 ± 8.32) [40], but our WEMWBS score were slightly lower. When a cut-off value of WEMWBS ≤ 40 was used to indicate a low level of well-being, 98 nurses were screened, accounting for 23.7%, which is similar to previous results. And when the cut-off value of GHQ-12 ≥ 3 was used to represent the presence of psychological symptoms, 159 nurses were found to have psychological symptoms, representing 38.5%. From the above data, it is clear that the overall health status of psychiatric nurses is poor, especially with negative symptoms.

Based on the dual-factor model of mental health, this study divided psychiatric nurses' mental health status into four groups, which also reflects the fact that well-being and psychiatric symptoms are not opposites of the same dimension, but independent but related structures. Our findings also found that the positive mental health group had the highest percentage (54.5%), indicating that half of the psychiatric nurses were in good psychological condition. The vulnerable group, which is defined as having low levels of well-being and psychological symptoms, represents 7% and traditional psychopathological models usually assume that this group is healthy, but in reality, the absence of psychological symptoms does not mean mental health, and thus this group can be neglected. In addition, the symptomatic but content group with significant psychological symptoms and high well-being accounted for 21.8%, suggesting that this group of patients can feel positive emotions despite their mental health problems. The trouble group with both psychological symptoms and lower well-being was 16.7% of the total population. Among these four groups, we found that the proportion of the positive mental health group was significantly lower among psychiatric nurses than among adolescents [41], college students [42], and normal individuals [43], yet the proportion of the troubled group was higher than the others. This is a strong indication that mental health of psychiatric nurses is not optimistic. It may be related to the high occupational stress of psychiatric nurses. Although no studies have directly compared the stress of adolescents, college students, normal groups, and psychiatric nurses, we believe that psychiatric nurses are the most stressed among these groups. Spearman's correlation analysis showed that occupational stress has a negative impact on mental health. Therefore, hospital managers can improve mental health by improving occupational stress management.

Our study confirmed the hypothesis that psychological resilience mediates the role of occupational stress and mental health in psychiatric nurses. However, due to the significant direct effect of occupational stress on mental health, a complete mediating effect was not obtained, which also serves as a side reminder of the multiple variables that can play a role in the relationship between occupational stress and mental health. Our study found that occupational stress can affect mental health not only directly, but also indirectly by affecting psychological resilience, which is consistent with the findings of Catabay et al. [44]. The findings of Lara-Cabrera et al. [45] pointed that psychological resilience mediated the role of occupational stress and depression, anxiety and psychological distress among nurses working. This also proves the stress theory [46], when psychiatric nurses experience more occupational stress, it negatively affects psychological resilience and to a certain extent reduces the level of resilience and sensitizes them, which in turn affects mental health. This also suggests that hospital managers can focus on the development and enhancement of psychological resilience in addition to reducing occupational stress in the process of improving mental health.

Our findings did not find that psychological resilience played a moderating role in occupational stress and mental health, which means that psychological resilience failed to mitigate the effects of occupational stress on mental health by reducing occupational stress. However, SONG et al. [47] indicated that psychological resilience moderated stress and depression in grass-roots civil servants. Anyan and Hjemdal [48] suggested that resilience also played a moderating role in stress and depression among adolescents. It may be related to the special nature of occupational stress in psychiatric nurses. Patients with mental disorders can suddenly exhibit dangerous behaviors such as emotional outbursts, violence and suicide, and put psychiatric nurses in a state of constant tenseness; In addition, the closed management of the ward can create a feeling of oppression and suffocation for nurses. Further, the frequent occurrence of workplace violence is causing secondary harm to psychiatric nurses. These are all signs of the special occupational stress of psychiatric nurses. This special occupational stress may have exceeded the buffering and regulating ability of psychological resilience, thus leading to the insignificant regulating effect of psychological resilience.

## Limitations and recommendations

It must be acknowledged that the study has some limitations. Firstly, a cross-sectional study was used and was unable to determine the causal relationship between variables. Secondly, although the participants in this study were from different levels of hospitals, there were only 17 nurses from primary hospitals, which was not representative enough. Finally, this study was conducted during the COVID-19 pandemic, which may have resulted in psychiatric nurses experiencing higher occupational stress and having lower mental health.

Despite these limitations of the present study, it also provides some information to the existing research. Based on the dual-factor model of mental health, this study described mental health from two perspectives of positive well-being and psychiatric symptoms and added mental health into the structural equation model, which makes the connotation of mental health of psychiatric nurses richer and more comprehensive. In addition, this study explored the mediating and moderating roles of psychological resilience in occupational stress and mental health of psychiatric nurses respectively and clarified the mechanisms. Mental health can be improved in the future in two different ways, one by reducing negative symptoms, such as reducing occupational stress, and the other by improving positive well-being, such as developing psychological resilience.

## Conclusions

Based on a dual-factor model of mental health, this study found that psychiatric nurses had a low overall state of mental health, with 54.5% positive mental health, 7% vulnerable, 21.8% symptomatic but content, and 16.7% completely troubled. Our findings also indicated that occupational stress was negatively associated with psychological resilience and mental health among psychiatric nurses, and that psychological resilience played a mediating role in occupational stress and mental health but did not play a moderating role. In the future, hospital managers need to pay attention to the vulnerable and symptomatic but satisfied individuals in addition to the completely troubled. Mental health can be improved both by reducing occupational stress through rationalizing work tasks, improving workplace violence management systems, and properly managing stress, as well as through psychological resilience interventions.

## Data Availability

All data generated or analyzed during this study are included in this published article or are available from the corresponding author on reasonable request.

## Abbreviations

CNSS: Chinese Nurses’ Stress Scale
CD-RISC: Connor-Davidson Resilience Scale
WEMWBS: Warwick-Edinburgh Mental Well-being Scale
GHQ-12: 12-Item General Health Questionnaire
PLS-SEM: Partial least square structural equation model
CR: Composite reliability
AVE: Average variance extracted
VIF: Variance inflation factors
SRMR: Standardized root mean residual
